# Effects of virtual reality training on decreasing the rates of needlestick or sharp injury in new-coming medical and nursing interns in Taiwan

**DOI:** 10.3352/jeehp.2020.17.1

**Published:** 2020-01-20

**Authors:** Szu-Hsien Wu, Chia-Chang Huang, Shiau-Shian Huang, Ying-Ying Yang, Chih-Wei Liu, Boaz Shulruf, Chen-Huan Chen

**Affiliations:** 1 Department of Surgery, Taipei Veterans General Hospital, Taipei, Taiwan; 2 Division of Clinical Skills Training, Department of Medical Education, Taipei Veterans General Hospital, Taipei, Taiwan; 3 School of Medicine, National Yang-Ming University, Taipei, Taiwan; 4 Bali Psychiatric Center, Ministry of Health and Welfare, New Taipei City, Taiwan; 5 Office of Medical Education, University of New South Wales Australia, Sydney, Australia; 6 Taipei Veterans General Hospital, Taipei, Taiwan; Hallym University, Korea

**Keywords:** Internship and residency, Needle stick injuries, Taiwan, Universal precautions, Virtual reality

## Abstract

**Purpose:**

Senior nursing and medical interns’ lack of familiarity and confidence with respect to practicing universal precaution for the prevention of occupational needlestick or sharp injuries may harm themselves. Trainees’ self-reported needlestick or sharp injury rate was known to be especially high during the first 2 months of internship in Taiwan. This prospective cohort study aimed to assess the effect of newly developed virtual reality (VR) game, which uses Gagne’s learning model to improve universal precaution for needlestick or sharp injury prevention and decrease the rates of needle stick or sharp injuries in new-coming medical and nursing interns in Taiwan.

**Methods:**

From 2017 to 2019, the VR system was developed and applied in training of 59 new-coming nursing and 50 medical interns. Occupational needlestick or sharp injury prevention was sought to be achieved through a game of right and wrong choices for safe or unsafe universal precaution behaviors.

**Results:**

In comparison with medical interns, a higher proportion of nursing interns had past experiences of deep occupational needlestick or sharp injury. Before VR training, the familiarity and confidence for needlestick or sharp injury prevention were higher among nursing interns than medical interns. Trainees with past experiences of deep needlestick or sharp injury exhibited better performance on the accuracy rate and time needed to complete 20 decisions than those without past experiences in VR practice. All trainees showed an improved performance after VR training. A high proportion of trainees reported that the VR-based training significantly decreased their anxiety about needlestick or sharp injury prevention.

**Conclusion:**

This self-developed VR game system using Gagne’s flow improved universal precaution for needlestick or sharp injury prevention and reduced the needlestick or sharp injury rates in the first 2 months of nursing and medical internship.

## Introduction

Occupational needlestick or sharp injuries (NSI) are defined as accidental percutaneous piercing wound caused by medical or laboratory equipment including needles, shredded intravenous cannulation devices, broken glass fragments, scalpels, lancets, pipettes or ampules, and injectors that can result in skin penetration injury. NSI are a major cause of occupational injuries for healthcare workers worldwide. The risk of occupational NSI among junior doctors was 3 times that of senior doctors. During their training period, 27%–40% of nursing and medical interns had a history of occupational NSI [1,2]. Usually, lectures are combined with simulation-based practice with mannequin in the skills lab to provide training on occupational NSI prevention before nursing and medical internship [3]. Even though most of the trainees report to have received training, the occupational NSI rate remained persistently high [4]. Accordingly, there is considerable room for the training tools and learning flows to be improved. In other words, more effective and feasible training tools and instructional flow are emerging to reduce the high occupational NSI rate among new nursing and medical interns.

The advancement in virtual reality (VR) technology allows learning to occur through immersive experiences. VR provides a controlled environment in which learners can navigate, manipulate and interact with the virtual objects and observe their effects in real time. Using VR technology in training can improve engagement and increase knowledge retention and satisfaction [5,6]. In medical education, game-based learning has gained popularity due to an increase in trainees’ sustained motivation and engagement [6]. Meanwhile, continuous evaluation and recording with apps make the outcomes of game-based learning testable and accessible [7].

Gagne’s flow is an information-processing model of mental events that occur when learners are presented with various learning objects. It highlights 9 specific instructional events, which correlate with crucial conditions of learning, and are arranged to enhance the learning process, improve session flow, and ultimately ensure that the learning objectives are comprehensively addressed [8]. However, not much research has been conducted on how VR, gaming, and apps can be appropriately integrated into Gagne’s flow for effective training.

This study aimed to evaluate the effectiveness of game-based VR training on universal precaution (UP) for occupational NSI prevention through Gagne’s flow. After using VR system, the familiarity, confidence, and anxiety of new-coming nursing and medical interns for NSI prevention were followed until 2 months after training.

## Methods

### Ethics statement

Ethical approval was granted by the ethics committee of the Taipei Veterans General Hospital, Taiwan (IRB approval no., 2018-07-030AC). All enrolled participants were informed about the importance and advantage of this intervention for their workplace safety and oral consent was obtained from all participants and questionnaire data were collected anonymously.

### Study design

This prospective and pre- and post-comparison study was done at Taipei Veterans General Hospital, a teaching hospital with 2,800 beds and 6,000 staffs, from September 2017 to September 2019.

### Participants

The study invited 59 new Chinese nursing and 50 new medical interns to join this new intervention at the beginning of their internship, typically within the first week. Participation was voluntary-base. They were informed that the refusal to join this new training did not affect their clinical performance grade. No control group was included in this study not to segregate them from new training course.

### Regular training model

In our system, after completion of their academic year and instructor-supervised clinical rotations, nursing and medical interns begin independent practice. As part of the regular training, the concept of UP for occupational NSI is formally introduced through lectures accompanied by slides, photos, videos, and post-class printed teaching materials, to the new-coming nursing and medical interns at the beginning of their independent practice.

### Development of the virtual reality game system

Monthly meetings were conducted to solicit feedback and to resolve ongoing issues and concerns. In addition to immersion and sense control, our game-based training was characterized by elements of curiosity, uncertainty, and surprise. In the VR environments, trainees faced 10 random scenarios for safe or unsafe behaviors with and without UPs for occupational NSI prevention (Supplement 1).

### Application of Gagne’s flow to a new model

**Fig. 1.**
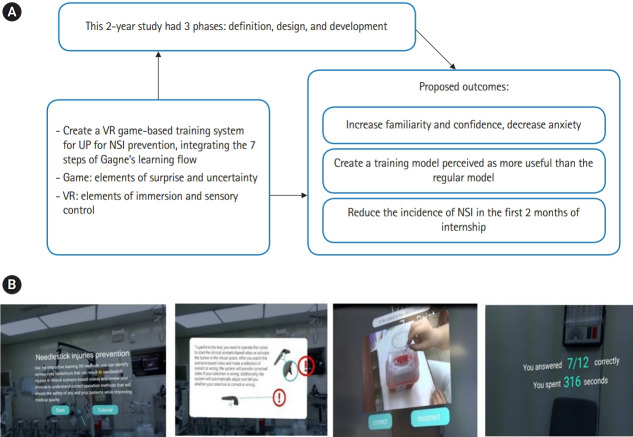
(A) The learning framework for new VR game-based training of UP for occupational NSI prevention. (B) Initial login page of the VR system, trainees chose safe or unsafe icon around the randomly appear 10 scenarios for either safe or unsafe behaviors. If the student make the wrong decision, App system will give comment wrong and provide correct answer. Conversely, the system will comment correct if the student makes correct decision. App subjectively evaluates and feedbacks performance of each student including accuracy rate and time need to complete 20 decisions, immediately. VR, virtual reality; UP, universal precaution; NSI, needlestick or sharp injuries.

Overall, the training integrated assessments of some specific steps in Gagne’s flow (Table 1). Each of Gagne’s instructional event was considered in conjunction with a variety of activities delineated to meet the diverse learning styles of students across clinical specialties [9-14]. In our study, the VR-based game system was incorporated into the modified steps in Gagne’s flow (Table 1). The VR system was designed using Unity 3D (Unity Technologies, San Francisco, CA, USA) incorporating physics, animations, and textures properties (Fig. 1B).

### Measurement tools

In Table 2, through the questionnaires that were incorporated into the VR system and the e-mail-based survey (follow-up), trainees were continuously assessed at different time points. After evaluation by 3 experts, it was found that the content validity index (CVI) of the Likert scale-based or dichotomous items in the questionnaire ranged from 0.76 to 0.9 (Table 2). In this study, the total scale-CVI of 0.84 indicated that experts considered the relevance of the scale for training objectives to be excellent. Similarly, the item-CVI values of the 6 items ranged from 0.77 to 0.87 (Table 2). Item 4 rated lower (0.77) than others indicating that it was considered as less reliable than the others by respondents.

### Statistical analysis

We used a series of paired Student t-tests on both samples to compare various pre-post data. Student t-tests or one-way analysis of variance was also used for comparison of pre-, post-, and follow-up data as well as various data between groups.

## Results

The items in the questionnaire, listed in Table 2, demonstrated good reliability (internal consistency) with a Cronbach α coefficient of 0.87. The data in Table 3 reveals no significant difference in the age range and mean age between nursing and medical interns. The proportion of female was higher among the nursing interns than the medical interns. In comparison with medical interns, a higher proportion of nursing interns reported having a previous experience of deep occupational NSI during instructor-supervised clinical rotations. Raw data were available from Dataset 1.

### Pre-and post-comparison study of virtual reality training

Before VR training, the familiarity and confidence levels in practicing UP for occupational NSI prevention were higher among nursing interns than medical interns. After VR practice, the familiarity and confidence levels were similar between the groups. Compared with pre-VR practice data, the rate of improvement in familiarity and confidence (familiarity: 356% versus 176%; confidence: 311% versus 134%) was higher among medical interns than nursing interns (familiarity: 95% confidence interval [CI], 1.145–1.448; P=0.004; confidence: 95% CI, 1.16–2.12; P=0.012) (Supplement 2). During the follow-up stage, i.e., 2 months later, the rate of improvement from pre-VR practice data (familiarity: 356% versus 176%; confidence: 311% versus 134%) remained unchanged from the post-VR practice stage. This result indicated good retention of the training effects on nursing and medical interns. A high proportion of nursing and medical interns (72% and 86%, respectively) reported that the VR-based training was more useful than regular training while 68% of nursing and 58% of medical intern reported that it significantly decreased their anxiety about occupational NSI prevention. During follow-up, the percentages of trainee that experienced more than 1 episode of occupational NSI in the first 2 months of internship was 31% (nursing interns) and 35% (medical interns) (Supplement 2). These data were significantly lower than our pre-intervention survey data (almost equal to 80%) obtained from a random sample of post-trained senior trainees, which had been mentioned in Supplement 1.

**Fig. 2.**
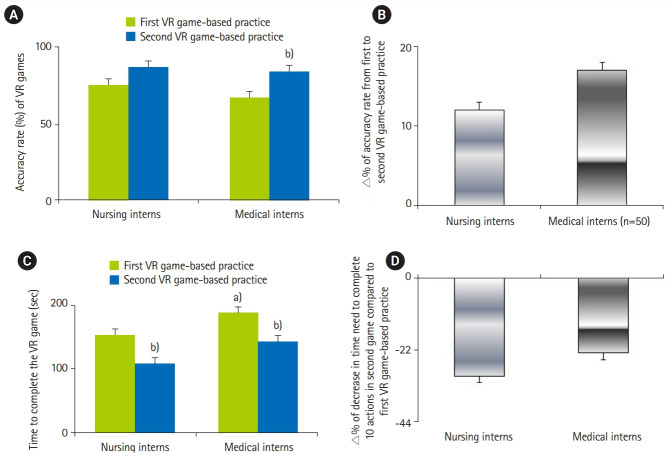
(A) Accuracy rate (%) of VR games between new nursing and medical interns. (B) ∆% of accuracy rate from first to second VR game-based practice. (C) Time to complete the VR game (sec). (D) ∆% of decrease in time need to complete 10 actions in second game compared to first VR game-based practice. VR, virtual reality. ^a)^P<0.05 vs. nursing group. ^b)^P<0.05 vs. first VR game-based practice.

**Fig. 3.**
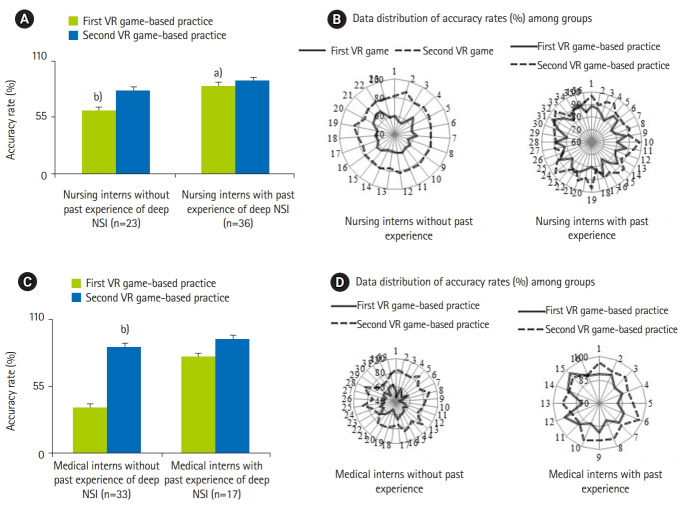
Comparison the mean and data distribution of accuracy rate (%) between nursing interns (A, B) or medical interns (C, D) with and without past experiences. VR, virtual reality; NSI, needlestick or sharp injuries. ^a)^P<0.05 vs. counterparts without past occupational NSI experience. ^b)^P<0.05 vs. first VR game-based practice.

In the first VR games, it was observed that the accuracy rate was similar between medical and nursing interns, but the medical interns took a relatively longer time (slower speed) than nursing interns to complete 10 decisions questions on safe or unsafe behaviors (95% CI, 1.3–2.71; P=0.02) (Figs. 2A, 3C). In the second round, both nursing and medical interns exhibited better performance, characterized by higher accuracy rate and shorter relative times compared to their first game performance (95% CI, 1.8–2.9; P=0.03) (Figs. 2A, 3C). In comparison with the first VR game, the improvement in the accuracy rate and time taken to complete the 10 decisions in the second VR game was more pronounced in the new-coming medical interns than the new-coming nursing interns (95% CI, 2.1–3.3; P=0.04.) (Fig. 2C, D).

**Fig. 4.**
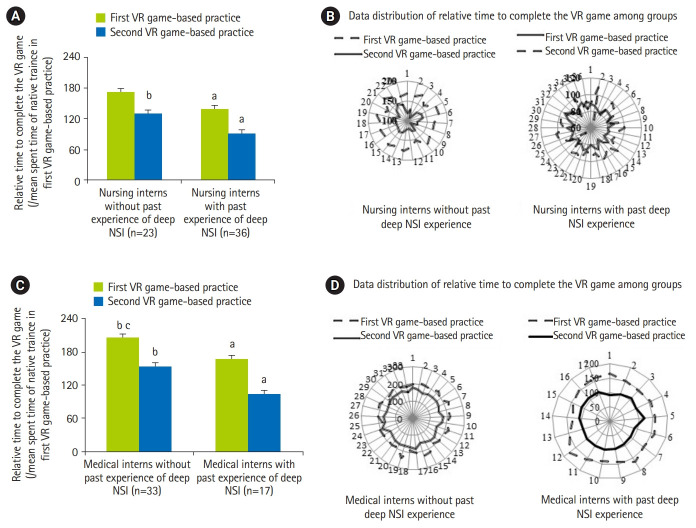
Comparison the mean and data distribution of the relative time to complete the VR game-based practice (/mean spent time of naive trainee in first VR game-based practice) between nursing interns (A, B) or medical interns (C, D) with and without past experiences. VR, virtual reality; NSI, needlestick or sharp injuries. ^a)^P<0.05 vs. counterparts without past occupational NSI experience. ^b)^P<0.05 vs. first VR game. ^c)^P<0.05 vs. nursing interns.

Figs. 3 and 4 show the mean and distribution of the accuracy rate and time taken to complete the decisions for the 2 groups. Notably, in the first VR game-based practice, nursing/medical interns with past deep occupational NSI experience had higher accuracy rate and needed lesser time to complete the 10 decisions than those without past deep occupational NSI experience (95% CI, 1.8–3.6; P=0.024) (Figs. 3, 4). In first the VR practice, the performance of nursing interns without past deep occupational NSI experience was significantly better than that of medical interns without past deep occupational NSI experience (95% CI, 1.8–2.9; P=0.037) (Fig. 3B, D). Significantly, the speed in making 10 decisions improved among all trainees during the first or second VR practice (Fig. 4).

## Discussion

### Key results

In our study, in comparison with medical interns, a higher proportion of nursing interns have had the past experiences of deep occupational NSI. Before VR training, the familiarity and confidence for NSI prevention were higher among nursing interns than medical interns. Trainees with past experiences of deep NSI exhibited better performance on the accuracy rate and time needed to complete 20 decisions than those without past experiences in VR practice. The performances of all trainees improved after VR training. A high proportion of trainees reported that the VR-based training significantly decreased their anxiety about NSI prevention.

### Interpretation and suggestion

Notably, our VR game training helps trainees experience the cognitive and integrative phases and finally proceed to the autonomous stage. In our study, the steps of Gagne’s flow of the course allow new medical and nursing interns to develop their UP skills for occupational NSI prevention through assessment, practice, and reflection. Meanwhile, the application-based assessment and feedback in our VR system increases the confidence of new trainees in this challenging skill. The VR training materials in our study also emphasized the principle of UP in operation room to avoid occupational NSI.

Failure to wear gloves is associated with various occupational NSI-related complications. Thus, the VR training materials in our study emphasized the importance of wearing gloves, masks, or goggles during risk practice.

Having a previous NSI experience was one of the risk factors for the occurrence of another injury [14]. So, it is reasonable to observe in our study that the occupational NSI rate was higher in trainees with a history of occupational NSI than in those without previous experience.

In our study, new nursing and medical interns reported that their anxiety regarding occupational NSI prevention decreased after the new VR game training. In our study, it is reasonable to find that the degree of improvement in performance (an accuracy rate and the time needed to complete 20 decisions) after VR game practice was higher among trainees with a past experience of deep occupational NSI than those without such experience. This result indicated that such a stressful experience will motivate learners to learn aggressively.

### Comparison with previous studies

The increasing incidence of blood exposure accidents due to the low familiarity and confidence levels among healthcare workers in practicing UP underscores the importance of training in occupational NSI prevention [10]. The costs of handling post-occupational NSI exposure evaluation, medical staff absence from work and hepatitis B and C treatment pose a significant economic burden on the healthcare system. Health workers should be trained regularly to reduce their risks of occupational NSI. It had been reported that regular preventive training can reduce the occupational NSI rate among nursing and medical students and registered nurses [10-12]. For new learners in the complex system, information should be presented in appropriate units for efficient processing via cognitive (assessment), integrative (practice), and autonomous (reflection) phases [10-12]. Healthcare workers who do not use personal protective equipment, such as gloves, masks, and goggles, during procedure were likely to have occupational NSI than their counterparts who wore protective gears [13]. Deep occupational NSI involving a visible puncture or cutting wound, especially with bleeding, is associated not only with physical issues, but also with anxiety, which can result in productivity reduction, absence from the job, work conflicts, and higher health care costs [15]. Naghayi et al. [15] reported that occupational NSI-related anxiety led to the absenteeism among many nurses and physicians. Occupational NSI results in a significant degree of anxiety among 12% of doctors.

### Limitation

Outcomes of training were evaluated mainly via a series of self-reported questionnaire. In the case of self-reported data, there may be a report bias, as trainees receiving specific training will be more likely to report behaviors consistent with what had been emphasized in training. Further, this study was conducted at a single medical center. Therefore, the application of this training model to other settings should be tested in the future. Despite these limitations, the positive results of this pilot study and the extent of current high occupational NSI rate among new nursing and medical interns suggest the need for continuous development and promotion of new feasible training models.

### Conclusion

This study investigates the effectiveness of our newly created VR game system in training nursing and medical interns on the practice of UP for occupational NSI at the beginning of their internship. Our new model significantly increased familiarity and confidence, and decreased anxiety of trainees for the prevention of occupational NSI. Especially, our self-developed VR game system reduced the NSI rates in the first 2 months of nursing and medical internship. Most of the trainees reported that the system is more useful than the regular model.

## Figures and Tables

**Table 1.** Seven steps of Gagne’s flow that addressing VR game-based training in our study

Step	Details of each step
First step: gain attention of trainees	Demographic data (age, gender, and past experience of deep occupational NSI) are collected. Part I introductory video, which introduce modes and risks of occupational exposure to body fluid and blood born pathogen, knowledge and skills of universal precaution for occupational NSI prevention, is provided for motivating students to learn.
Second step: inform trainees of objectives	Part II of introductory video is provided to increase awareness about the various safe or unsafe behaviors for occupational NSI prevention as mentioned in methods.
Third step: recall of prior learning	Complete the pre-VR questionnaire to assess familiarity and confidence on occupational NSI prevention.
Fourth step: provide learning guidance and feedback	Students wear headset and recognize randomly appear 10 scenarios of safe or unsafe behaviors for occupational NSI prevention. Using the VR handle, students chose safe or unsafe icon for either safe or unsafe behaviors. Both in first and second game (Fig. 2), if the student makes the wrong decision, App system will give comment wrong and provide correct answer. Conversely, the system will comment correct if the student makes correct decision.
Fifth step: assess performance	App in VR system subjectively evaluates and feedbacks performance of each student including accuracy rate and time for complete 10 actions in 2 VR game immediately.
Sixth step: eliciting performance	Students complete the post-VR questionnaire to assess their familiarity, confidence, and usefulness of the new model than regular model and degree of decrease anxiety.
Seven step: enhancing retention	Students get their performance and watch the introductory video again

VR, virtual reality; NSI, needlestick or sharp injuries.

**Table 2.** Questionnaire that incorporated in the VR system and e-mail-based survey (follow-up) for trainees’ self-assessment at different time points

Questions (Q)	Answers (A)	Pre-VR twice practice	Post-VR twice practice	2-month follow-up
Q1. Do you have the experience of deep occupational NSI during instructor-supervised clinical rotation?	A1. Please select your answer as 1 or 0 (1=yes, 0=no)	√		
Q2. Please assess your “familiarity” with safe behaviors for occupational NSI prevention.	A2. Please select your answer as 1, 2, or 3 (1: familiar with <30%, not very familiar; 2: 30%–70%, average; 3: >70%, very familiar)	√	√	√
Q3. Please assess your “confidence” on safe behaviors for occupational NSI prevention.	A3. Please select your answer as 0 or 1 (1: confidence in more than 80% of safe behavior; 0: have no confidence in more than 80% of safe behavior)	√	√	√
Q4. Do you agree that VR training is “useful” for teaching occupational NSI prevention than regular model?	A4. Please select your answer as 0 or 1 (0=no, 1=yes)		√	
Q5. Please evaluate the degree of this VR training “decrease anxiety” in practicing universal precaution for occupational NSI prevention.	A5. Please select your answer as 1, 2, 3, or 4 (1=not decrease, 2=mild decrease, 3=moderately decrease, 4=significantly decrease)		√	
Q6. Do you had more than 1 occupational NSI during the first 2 months of internship.	A6. Please select your answer as 1 or 0 (0=no, 1=yes)			√

VR, virtual reality; NSI, needlestick or sharp injuries.

**Table 3.** Comparison of the demographic data of new medical and nursing interns

Variable	Nursing interns (n=59)	Medical interns (n=50)
Age range (yr)	17–22	20–29
Mean age (yr)	19	22
Proportion of female (%)	50/59 (85)	26/50 (52)
% of trainees whose having a previous experience of deep occupational NSI during instructor-supervised clinical rotations	34	61

NSI, needlestick or sharp injuries.

## Footnotes

**Data availability**
Data files are available from https://doi.org/10.7910/DVN/GM6IE4 Dataset 1. Data file & data dictionary.

## Supplementary materials

Data files are available from https://doi.org/10.7910/DVN/GM6IE4

Supplement 1. Game-based training of new model.

Supplement 2. Comparison of the data of trainees self-assessed questionnaires incorporated in VR systems between groups.

Supplement 3. Audio recording of the abstract.
