# Supplementary material for: Effects of virtual reality training on decreasing the rates of needlestick or sharp injury in new-coming medical and nursing interns in Taiwan
Source: J Educ Eval Health Prof. 2020 Jan 20;17:1. doi: 10.3352/jeehp.2020.17.1 (PMC7054630; doi:10.3352/jeehp.2020.17.1)
Supplement: Supplementary file 2 — Supplement 1. Game-based training of new model. [file jeehp-17-01-suppl1.pdf]

Supplement 1. Game-based training of new model.

**Game-based training of new model**

The *safe* or *unsafe* behaviors were (1-2). not wearing gloves, a mask or goggles in risky scenario, (3).carelessly opening needle cap, (4). recapping of syringe needle after use, (5).dissembling needle and syringe after use, (6).needle perforated through cap, (7).bending needle before disposal, (8). transfer of blood into container on the rack, (9).breaking an ampoule or vial, (10).proper disposal into puncture proof container, (11).disposal of sharp containers that are 3/4 full, (12-14). proper handling of broken glass/intravenous catheter stylets/lancets, (15-17).proper exchanges of suturing needle, surgical knives and scalpels in the small space of an operating room, (18). avoid fumbling with sharps, and (19). avoid assembling/disassembling sharp, (20). cleaning sharp objects with forceps, etc. These safe or unsafe behaviors were developed from occupational NSI/SI literatures.

**Contents of questionnaires for self-assessment by trainees**

The items of questionnaire (Table 2) were included 1. Do you have [1 = yes, 0 = no] past experience of deep occupational NSI/SI during instructor-supervised clinical rotation? 2.Please assess your "*familiarity*" [1 = familiar with < 30% (*not very familiar*), 2 = 30-70% (*average*), 3 = > 70% (*very familiar*)] with *safe* behaviors for occupational NSI/SI prevention. 3.Please assess your "*confidence*" [confidence in more than 80% of *safe* behavior = 1 or have no confidence in more than 80% of safe behavior = 0] on practicing safe behaviors for occupational NSI/SI prevention. 4.Do you agree that VR training is "*useful*" (1 = yes/0 = no) for teaching occupational NSI/SI prevention than *regular* model? 5.Please evaluate the degree [4 = significantly decrease, 3 = moderately decrease, 2 = mild decrease, 1 = not decrease] of this VR training "*decrease your anxiety*" in practicing UP for occupational NSI/SI prevention 6. Do you had *more than one* occupational NSI/SI during the first 2-month of internship
