# Supplementary material for: Effects of virtual reality training on decreasing the rates of needlestick or sharp injury in new-coming medical and nursing interns in Taiwan
Source: J Educ Eval Health Prof. 2020 Jan 20;17:1. doi: 10.3352/jeehp.2020.17.1 (PMC7054630; doi:10.3352/jeehp.2020.17.1)
Supplement: Supplementary file 3 — Supplement 2. Comparison of the data of trainees self-assessed questionnaires incorporated in VR systems between groups. [file jeehp-17-01-suppl2.pdf]

Supplement 2. Comparison of the data of trainees self-assessed questionnaires incorporated in VR systems between groups

	Nursing interns (n = 59)			Medical interns (n = 50)		
% of "*very familiar*" with practicing *safe* behaviors with UP for NSI/SI prevention	pre-VR	post-VR	follow-up	pre-VR	post-VR	follow-up
	17/59 (29%)	47/59 (80%)#	40/59 (68%)	9/50	41/50 (82%)#	37/50 (74%)
		↑ 176% from pre-VR data	↑ 134% from pre-VR data	-18%	↑ 356% from pre-VR data**	↑ 311% from pre-VR data**
% of trainees agree that they "*have confidence*" (% of yes) in practicing *more than* 80% of *safe* behaviors with UP for NSI/SI prevention	pre-VR	post-VR	follow-up	pre-VR	post-VR	follow-up
	9/59 (15%)	50/59 (85%)#	49/59 (83%)	13/50 (26%)	43/50 (86%)#	45/50 (90%)
		↑ 467% from pre-VR data	↑ 453% from pre-VR data		↑ 231% from pre-VR data*	↑ 246% from pre-VR data*
% of trainees agree that VR training is "*useful*" (% of yes) than *regular* model	post-VR twice practice: 51/59 (86%)			post-VR twice practice: 36/50 (72%)		
Summative % of trainees reported that VR training "*significantly and moderately decrease their anxiety*"	post-VR twice practice: 40/59 (68%)			post-VR twice practice: 28/50 (58%)		
2-months follow-up % of "*have more than one* NSI/SI"	during the first 2 months of independent practice: 18/59 (31%)			during the first 2 months of independent practice:17/50 (34%)		

*p < 0.05 vs. nursing interns; #p < 0.05 vs. pre-VR data; [1 = "*not very familiar*"; 2 = average; 3 = very familiar; 1 = *familiar* with < 30%, 2 = 30-70%, 3 = > 70%] of safe behaviors for NSI/SI prevention; trainees self-assessed degree of VR practice on decreasing their anxiety on practicing UP for NSI/SI prevention is divided into significantly decrease (4), moderately decrease (3), mild decrease (2) and not decrease (1).
